# Ultrasonography of simple intratesticular cysts: a 13 year experience in a single centre

**DOI:** 10.1186/1746-1596-6-24

**Published:** 2011-03-24

**Authors:** Talal Al-Jabri, Saumya Misra, Zeshaan N Maan, Khalid Khan, Charles Coker, Phil Thompson

**Affiliations:** 1 Department of Surgery, East and North Hertfordshire NHS Trust, AL7 4JQ, UK; 2 Department of Urology, Princess Royal Hospital, Lewes Road, Haywards Heath, RH16 4EX, UK; 3 Department of Surgery, Colchester Hospital, Colchester, Essex, CO4 5JL, UK; 4 Department of Radiology, Princess Royal Hospital, Lewes Road, Haywards Heath, RH16 4EX, UK

## Abstract

**Objectives:**

Simple intratesticular cysts are being reported more commonly due to the wider use of scrotal ultrasonography however, their management remains unclear. Treatment has included enucleation, radical orchidectomy (over fear of an associated malignancy) and a more conservative approach with serial ultrasonography (if a neoplastic cyst is clearly ruled out). In view of the benign nature of such cysts, even serial ultrasonography may be unnecessary. We evaluate the presentation, diagnosis and management of ultrasound-detected simple intratesticular cysts over a 13-year period.

**Methods:**

Between May 1994 and August 2007, 24 men were found to have simple intratesticular cysts on scrotal ultrasonography. Records were analysed retrospectively to identify the clinicoradiologic findings and the management.

**Results:**

Median follow up was 29.5 months (range 4 - 108 months). Only one patient became symptomatic with a cyst which increased in size by 13 mm over 15 months. Orchidectomy performed at the patient's request confirmed a benign simple cyst.

**Conclusions:**

In our series, a significant change in size of the cyst with accompanying symptoms was observed in one case only. Asymptomatic patients with simple intratesticular cysts without associated features of bias towards malignancy can be discharged without need for further follow-up.

## Introduction

Intratesticular cysts, once thought to be a rarity, are now being reported with an increasing prevalence as a result of the wider use of scrotal ultrasound scanning [1]. Despite these reports, the management of intratesticular cysts remains unclear. Treatment has included enucleation and even radical orchidectomy over fear of the possibility of an associated malignancy. A more conservative approach with serial ultrasound scanning has been advocated if a clear distinction can be made between neoplastic and non-neoplastic testicular cysts [2-4]. However, in view of the benign nature of such cysts, even repeated ultrasound scanning may not be necessary and may be considered over-treatment. We review our experience of simple intratesticular cysts detected on ultrasonography over a 13 year period.

## Methods

**Figure 1:**
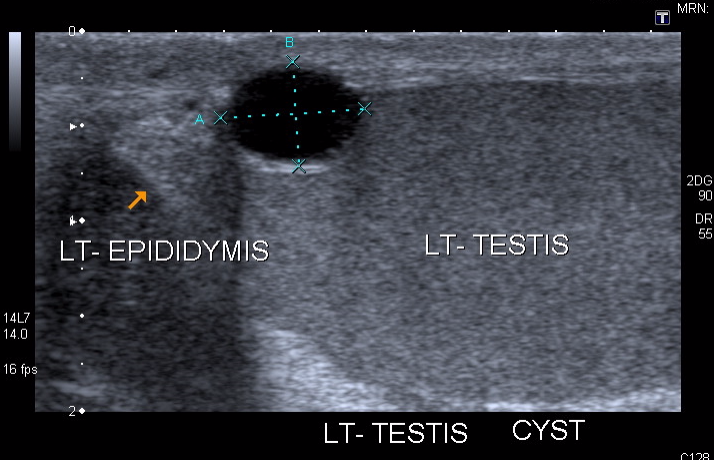
A 7 × 5 mm simple cyst in the upper pole of an otherwise normal testis - well circumscribed, anechoic area with smooth wall and posterior acoustic enhancement

The radiology department database was searched for scrotal ultrasound reports containing the words testicular cyst. Simple cysts were by definition, round anechoic lesions with a clear echogenic rim and through transmission with no alteration in the echotexture of surrounding testicular tissue (Figure 1) [4]. We identified 26 patients with this diagnosis between May 1994 and August 2007. Two patients were excluded from analysis: one patient's medical record was missing and the ultrasound description of the cyst in the second patient did not conform with the defined inclusion criteria of a simple cyst. Medical notes of the remaining 24 patients were reviewed retrospectively to ascertain the mode of presentation, clinicoradiologic findings and subsequent management. Follow-up scans were performed in our radiology department with a 5 MHz linear array transducer (Aloka SSD - 2000, Aloka Co Ltd, Tokyo, Japan) and a 7.5 MHz linear array transducer (Hitachi 6000, Hitachi Medical Corporation, Tokyo, Japan). Requirement for formal ethical approval was waived by the National Research Ethics Service.

## Results

The mean age of the 24 patients identified with cysts was 59 years (range 21 - 89 years). Patients were scanned for a variety of reasons (Table 1) and in most cases the intratesticular cyst was an incidental finding. Of the 24 patients at presentation, 19 (79%) had solitary cysts. One patient had multiple cysts, one had bilateral single cysts and three had two cysts each. Size of the cysts ranged from 2 mm to 44 mm and were present in the right (10), left (12) or both testes (2). All cysts except one were impalpable.

**Table 1:** Indications of scrotal ultrasound

Indications	Number of cases (n = 24)
Pain	5
Swelling	9
Both	2
TML follow up	3
Previous history of bilateral cysts	1
Loss of libido	1
Not known	3

Associated findings included epididymal cysts (15), hydroceles (4) and varicoceles (2). Three patients demonstrated features of testicular microlithiasis (TML). One patient had a contralateral atrophic testis, one patient showed changes consistent with previous hernia surgery, one patient had a solitary calcified focus and another had an echo poor area in the lower pole of the same testis. The latter patient was followed up for bilateral TML, and had a family history of testicular cancer.

The 24 patients had a variable number of ultrasound scans. Sixteen patients had two ultrasound scans and three patients had three ultrasound scans. Five patients were not available for follow-up (two had died of unrelated causes and three were not contactable). Of the patients who had three scans, two were followed up for TML and one patient had a repeat scan because of swelling and new pain.

At a median follow up of 29.5 months (range 4 - 108 months), there were no significant changes in the cysts except in one case. This patient had a palpable swelling but no pain initially. After the patient complained of pain, a repeat scan after 15 months showed the cyst had increased in size from 4.4 to 5.7 cm. An orchidectomy was performed at the patient's request and histology confirmed a cystic area lined with flattened non-ciliated epithelium with clear serous fluid and no signs of malignancy.

## Discussion

Testicular cysts were once considered rare but with increasing use of scrotal ultrasound, prevalence would appear to be increasing [1]. Leung et al scanned 40 normal male volunteers and found simple cysts in 8% [5]. Gooding also found testicular cysts present in 9.8% of 307 normal men undergoing high resolution scrotal ultrasound scanning [6]. Simple intratesticular cysts have a pathognomonic echo pattern and high resolution ultrasound permits clear distinction between simple intratesticular cysts and other cystic lesions like epidermoid cysts and cystic testicular tumours [7].

In a large series of 847 men, Hamm et al found cystic tumors in 16 of the 34 men with cysts in the testes [1]. Nearly all presented with a palpable mass and 75% had solid elements on scanning. Of the 18 non-neoplastic cysts, 13 were simple intratesticular cysts which were all accidentally discovered, impalpable and had classical sonographic characteristics. Testicular cysts range in size from 2 mm to 2 cm [6]. While the majority are found within the inner substance of the testis, those that are peripherally oriented may be suspected on physical examination by palpation of a focal, non-tender mass [8]. In our series, only one 44 mm cyst was palpable and none of the cysts were symptomatic.

Management of simple intratesticular cysts is controversial. Surgical treatments have ranged from simple enucleation to radical orchidectomy [2,3]. An organ preserving operation for a small cyst can be a difficult task and intraoperative localization can sometimes be done only via intraoperative ultrasound [3]. In most series where intratesticular cysts have been reported, when preoperative ultrasonography had been performed, the sonographic diagnosis of simple cyst was confirmed in the surgical specimen [1,3,9]. Therefore conservative management with repeat ultrasound scanning was felt appropriate. However, a follow up strategy has not been clearly defined. Hobarth et al followed 17 patients with intratesticular cysts with an intensive regime (consisting of an initial chest X ray, sonography of the retroperitoneal space, tumor marker identification) for an average of 29.3 months (range 1 - 108) and found no change in the cysts [4]. More recently, Shergill et al reported their experience with follow up of simple testicular cysts by USS in a series of 24 patients [10]. The cysts did not change significantly and none of the patients required surgical intervention. The frequency of scans and reasons for prolonged follow up (median 32 months) are however not clear and it appears that most patients had repeated ultrasound scans because of anxiety. Hatsiopoulou et al suggested following up asymptomatic cysts but did not say for how long [11].

Furthermore, the average age at presentation in most series is between 60 and 65 years (our average was 59 years), a population in whom germ cell malignancy is admittedly less common [6,10]. Repeated ultrasound scanning therefore may not be justified.

Our review revealed that most patients had two ultrasound scans and were discharged from further follow up when the second scan showed no change. None of the patients returned with problems as a consequence of the cysts. Two patients died of unrelated causes. The only patients who were followed up longer were those with TML and the lone patient who represented with a palpable swelling and pain. Chronic scrotal pain from simple cysts although rare, has been reported [12]. In our series there was only one patient who had a palpable painless swelling at presentation, which later became painful. The benign nature of the cyst was confirmed at histology.

It has been suggested that testicular cysts arise from remnants of Mullerian or Wolfian ducts, since such cysts have been reported in children [9]. Other possible etiologies include trauma and inflammation, which can cause occlusion of the spermatic ducts with subsequent ectasia and cystic alterations in the rete testis [1,13]. There was no history of trauma or inflammation in our patients and therefore the etiology of the cysts in these patients remains unclear.

We accept that our study being a retrospective review has limitations. Patients were scanned by different radiologists over a period of time who gave recommendations, based on their personal opinion. Follow-up data was complete for all but five patients. Our main findings concerning simple intratesticular cysts are however consistent with the literature, in that they are almost always accidentally discovered, are impalpable and are not generally associated with symptoms. Very occasionally cysts can enlarge and cause pain, as shown by the lone example in our study. None of the patients required surgery for malignant transformation and most patients were reassured and discharged after repeat scanning.

## Conclusions

In our case series of 24 patients with simple intratesticular cysts, all except one cyst remained unchanged. We believe that symptomatic intratesticular cysts should be watched for a period of time to ensure that there is no change in size or echogenecity, but asymptomatic patients with simple intratesticular cysts without associated features of bias towards malignancy can be discharged without need for further follow-up.

## Abbreviations

TML: Testicular microlithiasis; USS: Ultrasound scan.

## Competing interests

The authors declare that they have no competing interests.

## Authors' contributions

TAJ and SM devised the project, collected the data and wrote the paper. KK and PT performed the

scans and helped edit the manuscript. ZNM, TAJ and CC edited the manuscript. All authors have read and approved the manuscript.
